# Supplementary material for: Qki activates Srebp2-mediated cholesterol biosynthesis for maintenance of eye lens transparency
Source: Nat Commun. 2021 May 21;12:3005. doi: 10.1038/s41467-021-22782-0 (PMC8139980; doi:10.1038/s41467-021-22782-0)
Supplement: Supplementary file 1 — Supplementary Information [file 41467_2021_22782_MOESM1_ESM.pdf]

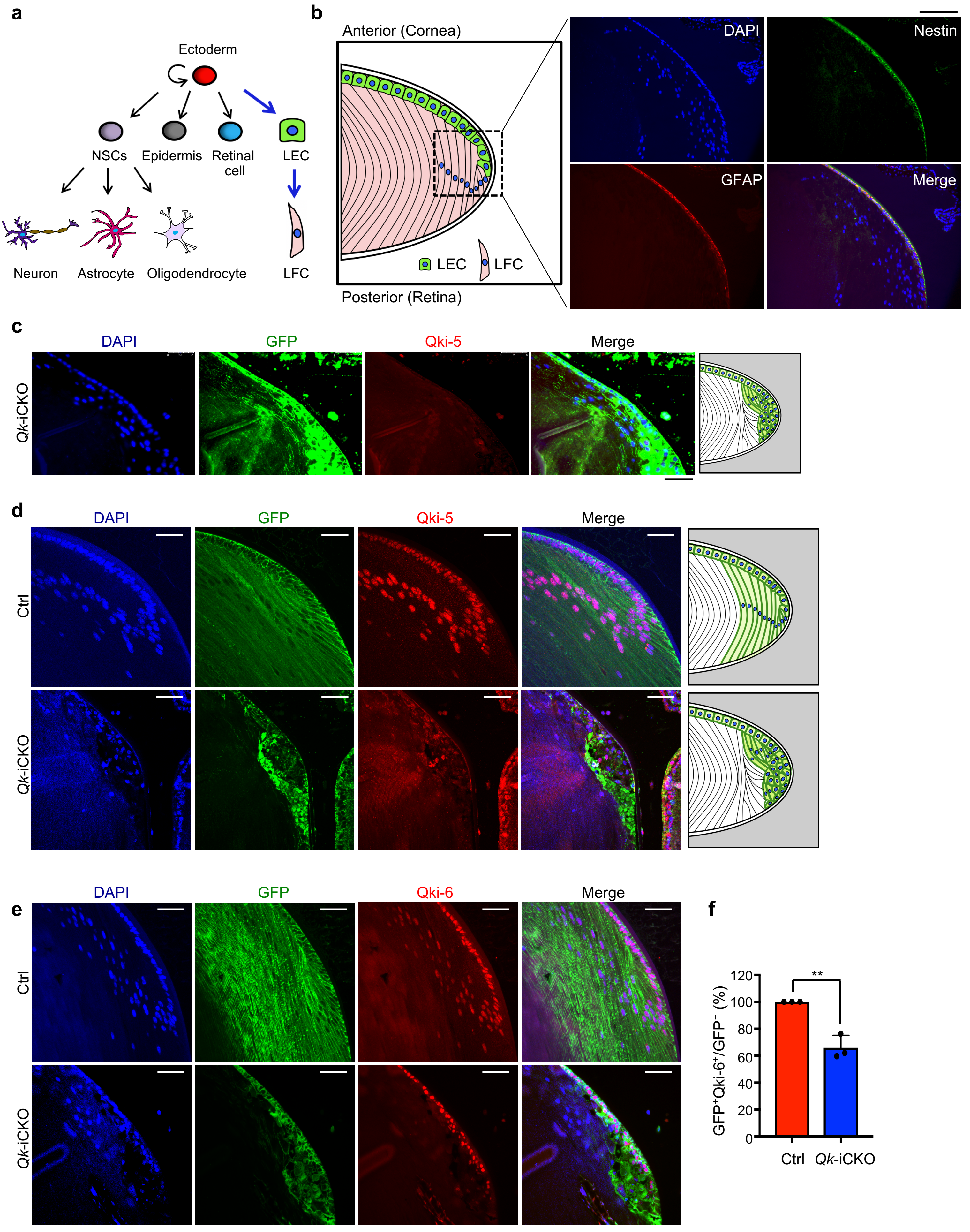

Shin et al Supplementary Fig. 1

Supplementary Fig. 1: Establishment of the mouse model to investigate the role of Qki in eye lens. a Schematic of the ectodermal lineage during early development. Blue arrow indicates eye lens cell lineage differentiated from the ectoderm origin. LEC: Lens epithelial cell. LFC: Lens fiber cell. **b** Representative immunofluorescent stains of lens cells (within the dotted box in the schematic of the eye lens structure) in paraffin-embedded sections of ocular tissue for Nestin (green) and GFAP (red) (scale bar: 200 μm) at P19. DAPI (blue): nuclei. The result was replicated three times. **c** Representative immunofluorescent stains of lens cells in paraffin-embedded sections of ocular tissue from *Qk*-iCKO mice for GFP (green) and Qki-5 (red) (scale bar: 50 μm) at P19. DAPI (blue): nuclei. The images are identical with the images from *Qk*-iCKO in **Fig. 1a** except for the stronger GFP exposure to trace all the GFP^+ lens cells. The result was replicated three times. **d** Representative immunofluorescent staining of lens cells in paraffin-embedded sections of ocular tissue from control and *Qk*-iCKO mice for GFP (green) and Qki-5 (red) (scale bar: 50 μm) at P19. DAPI (blue): nuclei. Schematic at the far right depicting the morphological impairment of eye lens structure upon Qki loss in *Qk*-iCKO mice compared to the control mice. Images taken by confocal microscopy. The result was replicated three times. **e** Representative immunofluorescent stains of lens cells in paraffin-embedded sections of ocular tissue from control and *Qk*-iCKO mice for GFP (green) and Qki-6 (red) (scale bar: 50 μm) at P19. DAPI (blue): nuclei. Images taken by confocal microscopy. **f** Quantification of GFP^+Qki6^+ cells among all GFP^+ cells in the eye lens tissue represented in **e**. n = 3 mice/group. The results are presented as means with SD. $p = 0.002887$. $^{**}p < 0.01$ (two-tailed unpaired *t*-test).

a

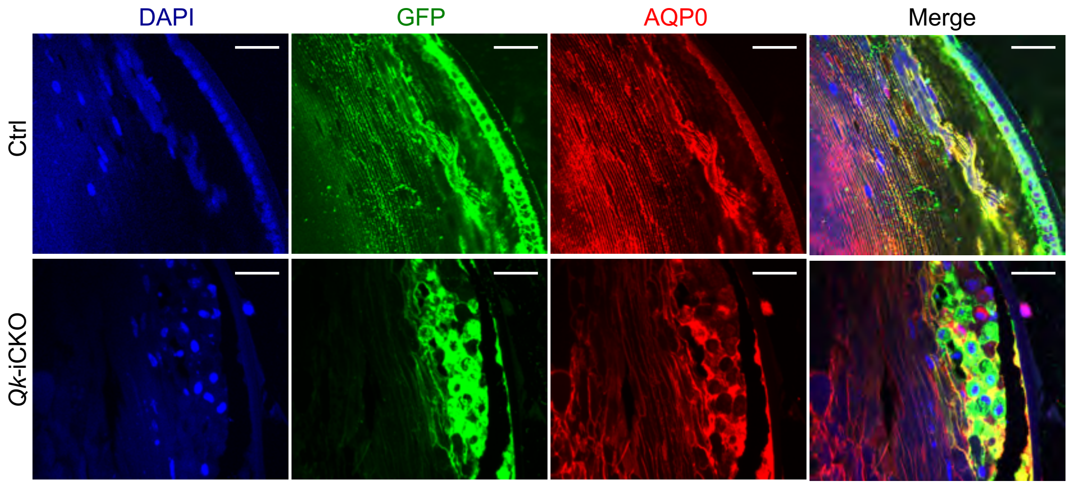

Supplementary Fig. 2: Impairment of lens membrane structure in Qki-depleted lens tissue.

a Representative immunofluorescent stains of lens cells in paraffin-embedded sections of ocular tissue from Ctrl and *Qk*-iCKO mice for GFP (green) and AQP0 (red) (scale bar: 50 μm) at P19. DAPI (blue): nuclei. Images taken by confocal microscopy. All the experiments were replicated three times in the lab.

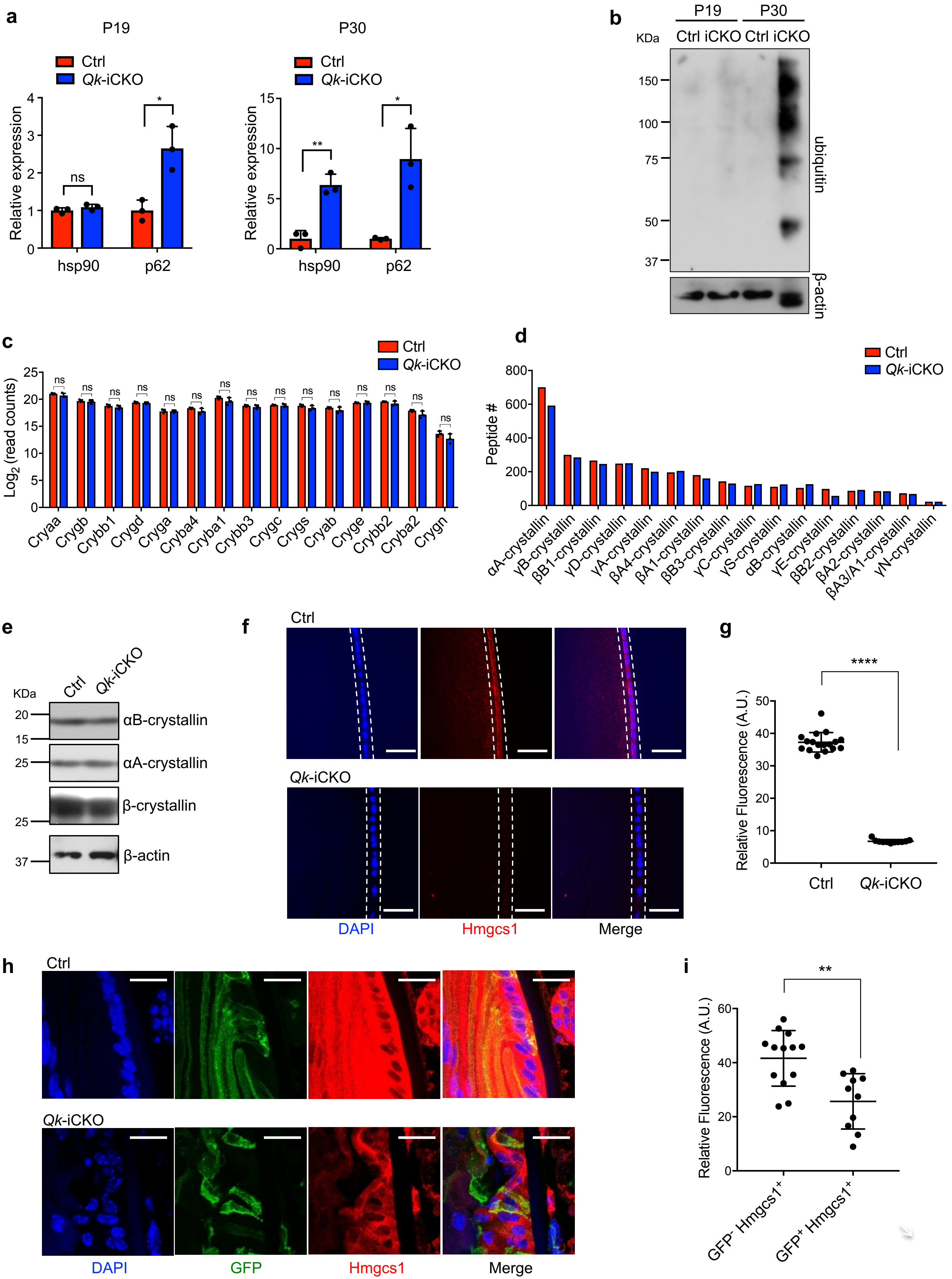

Shin et al Supplementary Fig. 3

Supplementary Fig. 3: Comparison of the expression level of proteostatic markers, crystallin isoforms, and Hmgcs1 in the lenses of control and *Qk*-iCKO mice. a Quantification of proteostatic markers, hsp90 and p62 in isolated Ctrl (n = 3) and *Qk*-iCKO (n = 3) lenses at P19 and P30. $p = 0.2191$ (hsp90, P19); 0.01143 (p62, P19); 0.002429 (hsp90, P30); 0.01096 (p62, P30), $^{*}p < 0.05$; $^{**}p < 0.01$; ns = not significant ($p \geq 0.05$) (two-tailed unpaired *t*-test). The results are presented as means with SD. **b** Immunoblots of proteostatic marker, ubiquitin in isolated Ctrl and *Qk*-iCKO lenses at P19 and P30. β-actin: loading control. **c** Raw read counts in RNA-seq data for crystallin isoforms in lenses isolated from Ctrl (n = 3) and *Qk*-iCKO (n = 3) mice at P17-19. $p = 0.2550$ (Cryaa); 0.6485 (Crygb); 0.3587 (Crybb1); 0.5985 (Crygd); 0.9788 (Cryga); 0.1548 (Cryba4); 0.2052 (Cryba1); 0.4050 (Crybb3); 0.5845 (Crygc); 0.2291 (Crygs); 0.2721 (Cryab); 0.9551 (Cryge); 0.2290 (Crybb2); 0.1341 (Cryba2); 0.2052 (Crygn), ns = not significant ($p \geq 0.05$; two-tailed unpaired *t*-test). The results are presented as means with SD. **d** Peptide numbers (#) for crystallin isoforms in lenses isolated from Ctrl and *Qk*-iCKO mice at P19 according to mass spectrometry. **e** Immunoblots of lenses isolated from Ctrl and *Qk*-iCKO mice at P19 for detection of αB-crystallin, αA-crystallin, and β-crystallin. β-actin: loading control. **f** Representative immunofluorescent stains of lens cells from paraffin-embedded sections of ocular tissues obtained from Ctrl and *Qk*-iCKO mice at P19 for Hmgcs1 (red). DAPI (blue): nuclei. Scale bar: 40 μm. **g** Quantification of the Hmgcs1^{+} signal (A.U.) in the regions between the white dotted lines in (**f**). $^{****}p < 0.0001$ (two-tailed unpaired *t*-test). The results are presented as means with SD. **h** Representative immunofluorescent stains of lens cells from paraffin-embedded sections of ocular tissues obtained from Ctrl and *Qk*-iCKO mice at P19 for GFP (green) and Hmgcs1 (red). DAPI (blue): nuclei. Scale bar: 40 μm. **i** Quantification of GFP^{-}Hmgcs1^{+} and GFP^{+}Hmgcs1^{+} signals (A.U.) in the *Qk*-iCKO lens cells in (**h**). $p = 0.0014$,

**$p < 0.01$ (two-tailed unpaired *t*-test). The results are presented as means with SD. All the experiments were replicated three times in the lab.

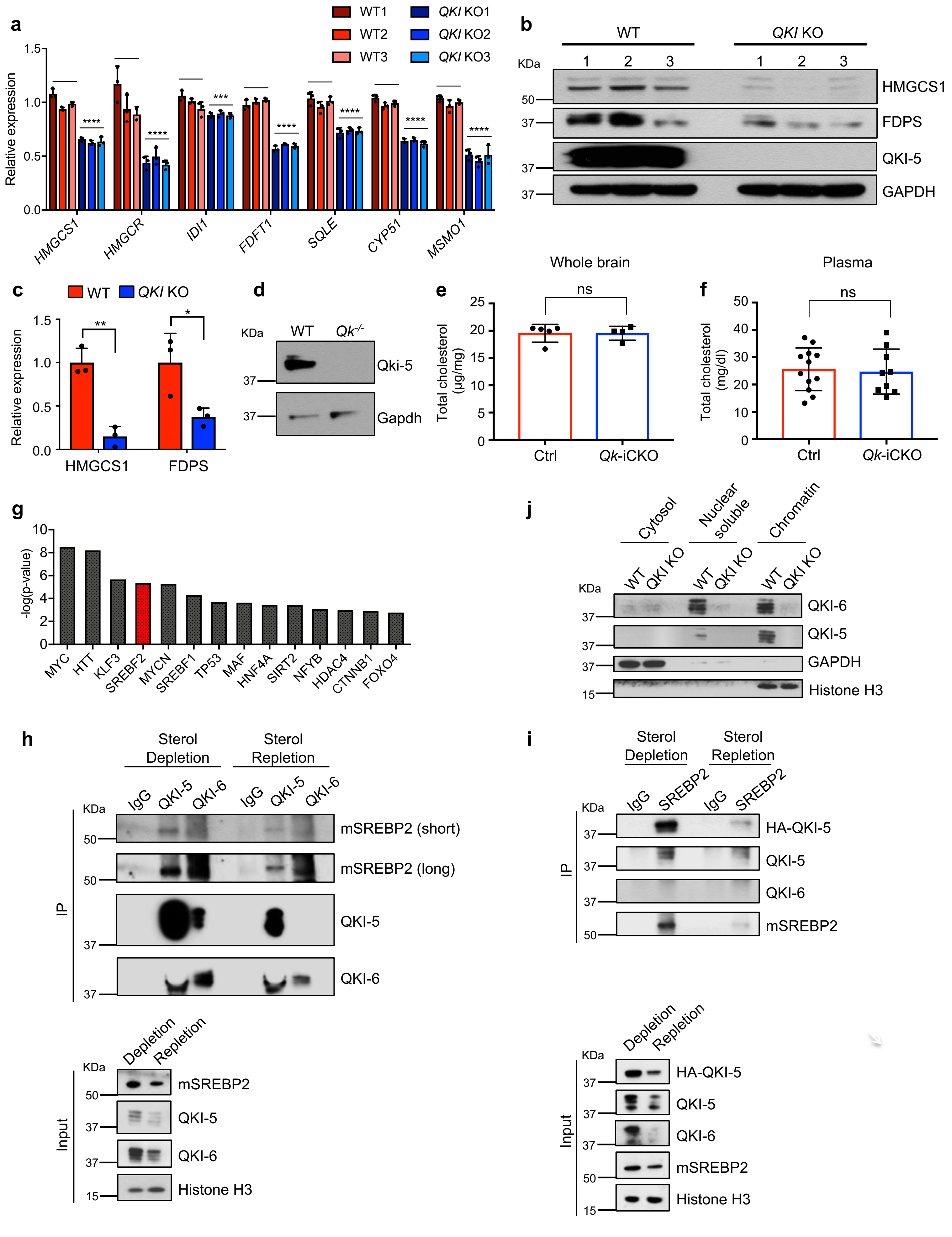

Shin et al Supplementary Fig. 4

Supplementary Fig. 4: A collaboration between QKI and mature SREBP2 in regulation of cholesterol biosynthesis pathway. a qPCR analysis of cholesterol biosynthesis genes in WT (n = 3) and *QKI* KO HLE-B3 (n = 3) cells. $p = 0.0000000001092$ (HMGCS1); 0.0000002293 (HMGCR); 0.0001723 (IDI1); 0.000000000000005 (FDFT1); 0.000000001438 (SQLE); 0.000000000000134 (CYP51); 0.0000000000173 (MSMO1), $^{***}p < 0.001$; $^{****}p < 0.0001$ (two-tailed unpaired *t*-test). The results are presented as means with SD. **b-c** Immunoblots and quantification of WT (n =3) and *QKI* KO HLE-B3 (n = 3) cells for detection of cholesterol biosynthesis enzymes (HMGCS1 and FDPS) and immunoblots of QKI-5. GAPDH: loading control. The results in **c** are presented as means with SD. $p = 0.001859$ (HMGCS1); 0.03741 (FDPS) $^{*}p < 0.05$; $^{**}p < 0.01$ (two-tailed unpaired *t*-test). **d** Immunoblots of WT and $Qk^{-/-}$ NLPCs for detection of Qki-5. GAPDH: loading control. The result was repeated three times. **e-f** Quantification of total cholesterol levels in (**e**) whole brain and (**f**) blood (plasma) from the control and *Qk*-iCKO mice at P19. (**e**) $p = 0.9992$. (**f**) $p = 0.8126$. ns = not significant ($p \geq 0.05$; two-tailed unpaired *t*-test). The results are presented as means with SD. (**e**) Ctrl: n = 5 mice/group, *Qk*-iCKO: n = 4 mice/group (**f**) Ctrl: n = 12 mice/group, *Qk*-iCKO: n = 9 mice/group. **g** The top enriched upstream transcription regulators of the genes expressed at lower levels in *Qk*-iCKO mice than in Ctrl mice according to RNA-seq data ($p < 0.05$; two-tailed Wald test) ranked according to *p*-value derived by right-tailed Fischer's exact *t*-test. **h-i** Co-IP of HLE-B3 cells with (**h**) anti–QKI-5 and anti-QKI-6 antibodies blotting with anti-SREBP2, anti–QKI-5, and anti–QKI-6 (**i**) anti-SREBP2 antibody blotting with anti-HA, anti-QKI-5, anti-QKI-6, and anti-SREBP2 antibodies. Sterol repletion with 25-hydroxycholesterol (50 µg/mg) for 3 hours. mSREBP2: mature SREBP2. Histone H3: loading control. The results were repeated three times.

j Immunoblots for detection of QKI-5 and QKI-6 in subcellular fractions of WT and *QKI* KO HLE-B3 cells. GAPDH: cytosol; Histone H3: chromatin. The result was repeated three times.

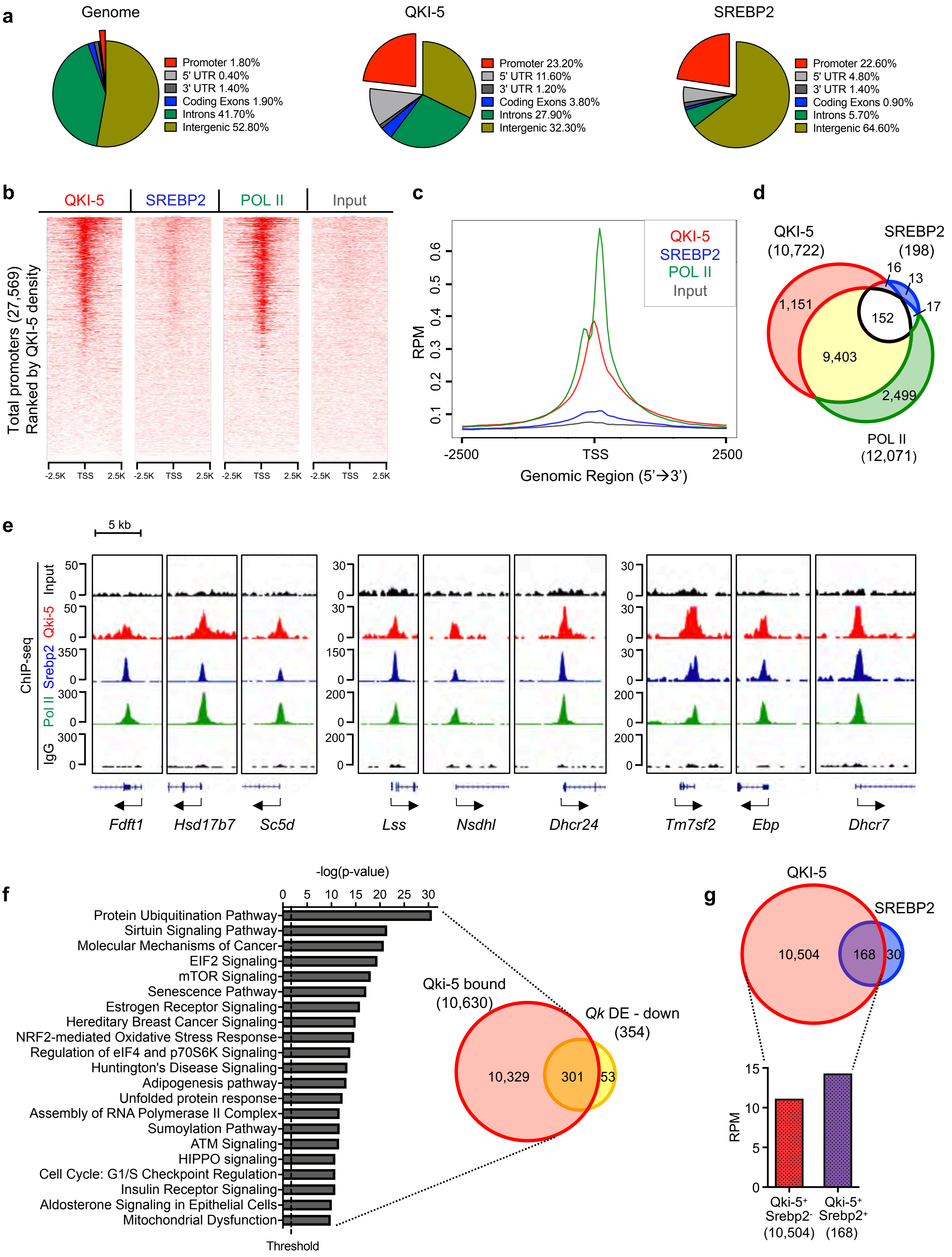

Shin et al Supplementary Fig. 5

Supplementary Fig. 5: ChIP-seq data of HLE-B3 cells and NLPCs. a Genomic annotation of genome or QKI-5– and SREBP2-binding sites from ChIP-seq in HLE-B3 cells. Promoters are confined to the regions ±2 kb from the TSS. **b** Heat map of QKI-5, SREBP2, and POL II ChIP-seq and input signals within ±2.5 kb from the TSS regions for all promoters (n = 27,569) in HLE-B3 cells ranked by QKI-5 density. **c** Signal plots showing RPM for QKI-5, SREBP2, and POL II ChIP-seq data and input ±2.5 kb from the TSS of all promoter regions (n = 27,569) in HLE-B3 cells. **d** Venn diagram showing the overlapping promoters bound by QKI-5, SREBP2, and POL II in HLE-B3 cells. Promoter defined by TSS ± 2 kb. **e** UCSC Genome Browser snapshot of the promoter regions of cholesterol biosynthesis genes in Qki-5, Srebp2, and Pol II ChIP-seq data in NLPCs. Input and rabbit IgG are used as a control. **f** IPA of the genes bound by Qki-5, but not downregulated upon QKI depletion in both NLPCs and HLE-B3 cells according to RNA-seq analysis (n = 10,329) ranked according to significance (*p*-value) ($p < 0.05$; right-tailed Fischer's exact *t*-test). DE: differentially expressed. **g** Comparison of the average RPM values of the QKI-5^{+}SREBP2^{-} (n = 10,504) and QKI-5^{+}SREBP2^{+} (n = 168) ChIP-seq binding events within ±0.5 kb from the TSS in HLE-B3 cells.

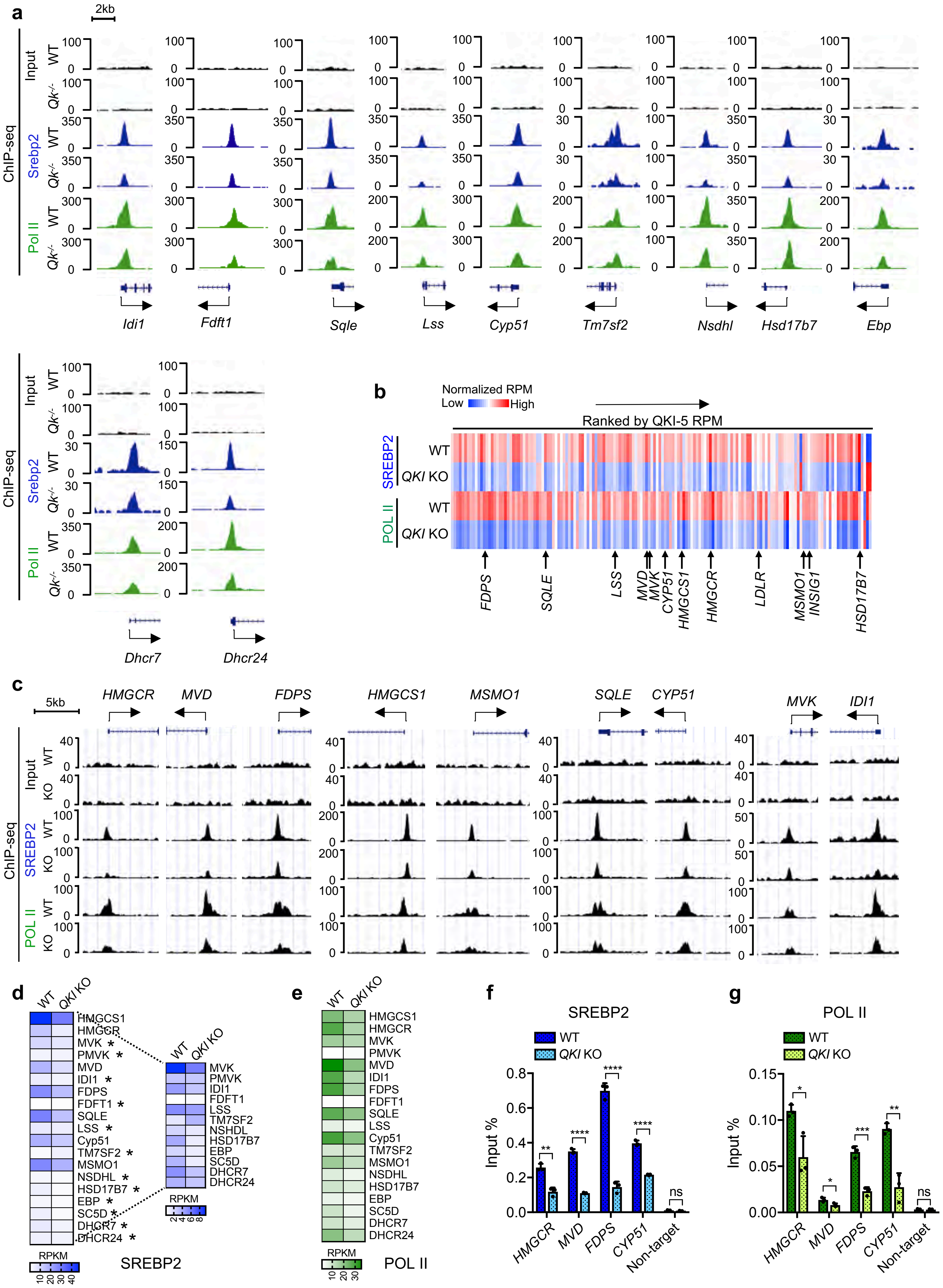

Shin et al Supplementary Fig. 6

Supplementary Fig. 6: Decreased occupancy of SREBP2 and POL II upon *QKI* loss in NLPCs and HLE-B3 cells. **a** UCSC Genome Browser snapshot of the promoter regions of cholesterol biosynthesis genes from ChIP-seq of WT and $Qk^{-/-}$ NLPCs. **b** Heat map of normalized RPM (RPM/average of RPM^{WT} and $RPM^{QKI\ KO}$) in WT and *QKI* KO HLE-B3 cells in SREBP2 and POL II ChIP-seq data ranked by QKI-5 ChIP-seq read densities (RPM). Arrows indicate genes involved in cholesterol metabolism. **c** UCSC Genome Browser snapshot of the promoter regions of cholesterol biosynthesis genes from ChIP-seq of WT and *QKI* KO HLE-B3 cells. **d-e** Heat maps comparing the RPM (±0.5 kb from the TSS) in the promoter regions of cholesterol biosynthesis genes in WT and *QKI* KO HLE-B3 cells in SREBP2 and POL II ChIP-seq data. Genes with asterisk (*) in SREBP2 ChIP-seq were shown as a separate heat map. **f-g** ChIP-qPCR analysis of the promoter regions of *HMGCR*, *MVD*, *FDPS*, and *CYP51* in WT (n = 3) and *QKI* KO (n = 3) HLE-B3 cells. (**f**) $p = 0.001682$ (*HMGCR*); 0.000008146 (*MVD*); 0.00006156 (*FDPS*); 0.00004750 (*CYP51*); 0.07977 (Non-target). (**g**) $p = 0.02171$ (*HMGCR*); 0.03845 (*MVD*); 0.0006224 (*FDPS*); 0.002560 (*CYP51*); 0.8780 (Non-target). $^{*}p < 0.05$; $^{**}p < 0.01$; $^{***}p < 0.001$; $^{****}p < 0.0001$; ns = not significant ($p \geq 0.05$) (two-tailed unpaired *t*-test). The results are presented as means with SD.

a

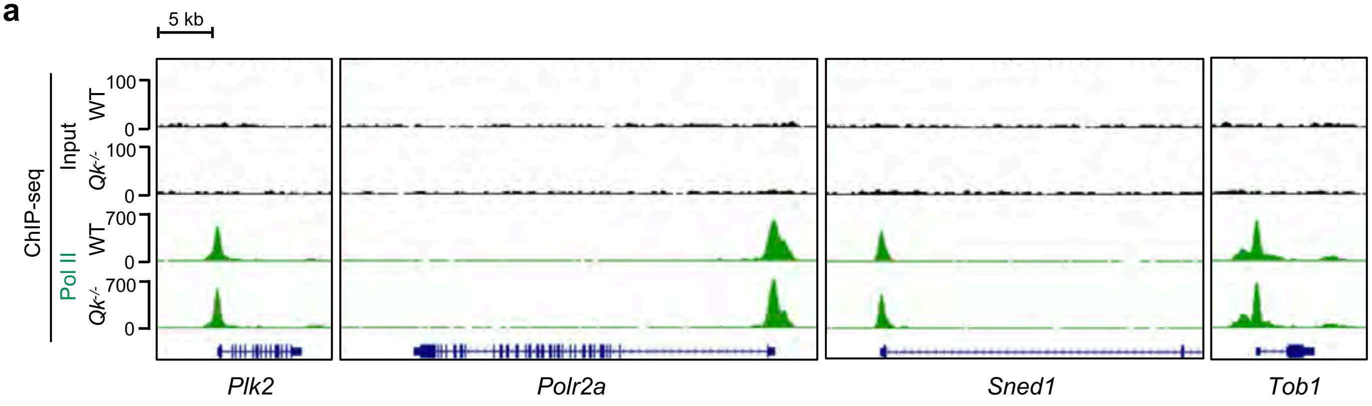
5 kb
ChIP-seq
Input
Pol II
WT
Qk-/-
100
0
700
Plk2
Polr2a
Sned1
Tob1

b

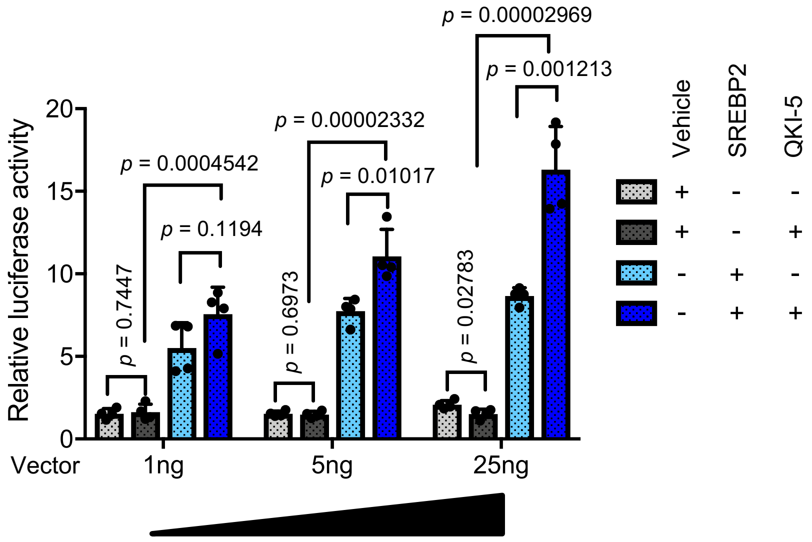
Relative luciferase activity
20
15
10
5
0
Vector
1ng
5ng
25ng
p = 0.7447
p = 0.0004542
p = 0.1194
p = 0.6973
p = 0.00002332
p = 0.01017
p = 0.02783
p = 0.00002969
p = 0.001213
Vehicle
SREBP2
QKI-5
+ - -
+ - +
- + -
- + +

Supplementary Fig. 7: QKI-5 enhances the transcriptional activity of SREBP2. a UCSC Genome Browser snapshot of the genes unchanged with Pol II occupancy from Pol II ChIP-seq data in NLPCs. **b** Bar graph showing the expression of SRE luciferase reporter in HEK293FT cells with ectopically expressed empty vector, SREBP2, or QKI-5 in a dose-dependent manner (1ng, 5ng, and 25ng). *p*-value is indicated for each group in the figure accordingly (two-tailed unpaired *t*-test). The results are presented as means with SD. n = 4/group.

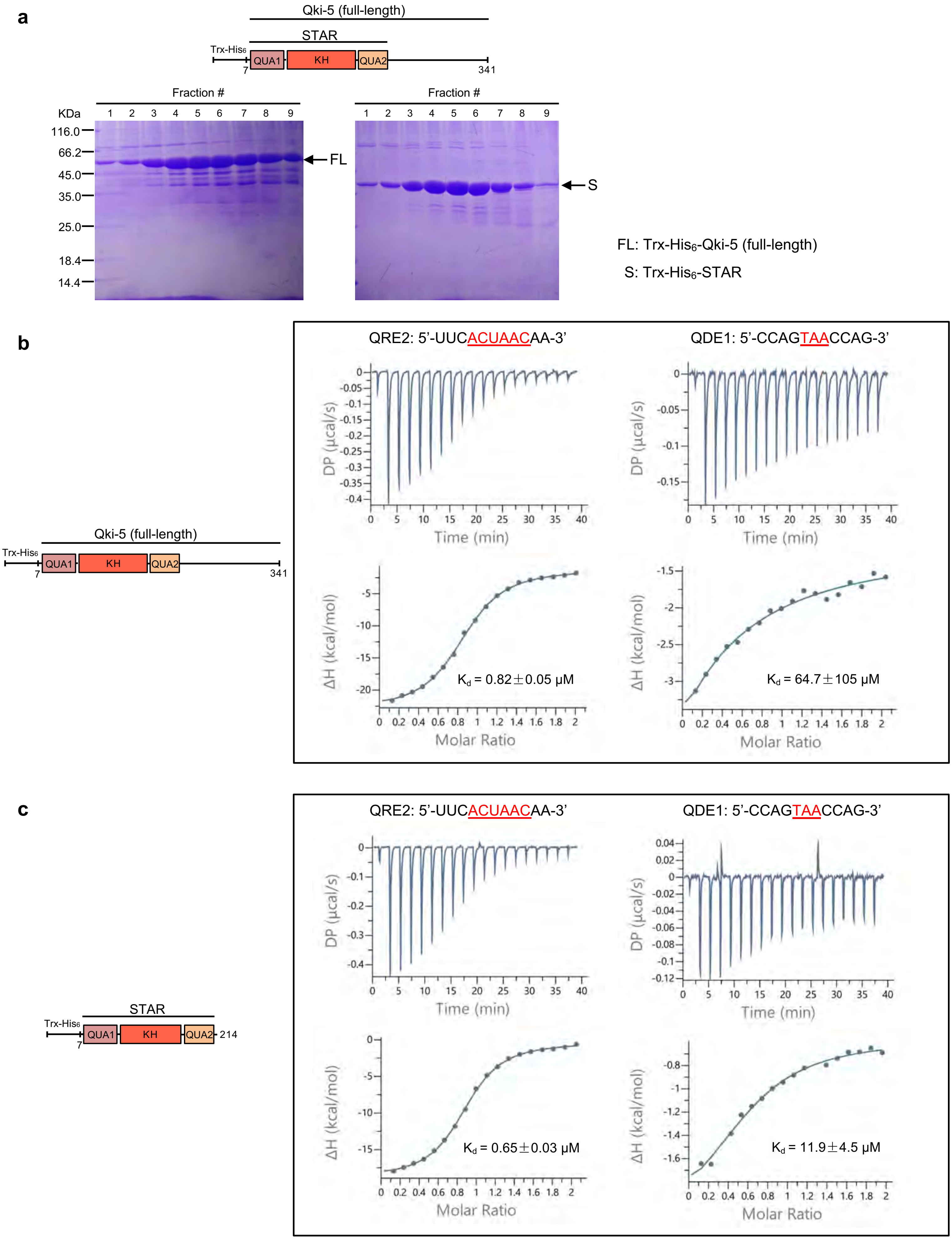

Shin et al Supplementary Fig. 8

Supplementary Fig. 8: Qki-5 interacts with ssDNA according to ITC assay. a Schematic of full-length and the STAR domain of Qki-5 and Coomassie blue-stained gels of purified proteins (FL and S) in the stepwise fractionation procedure using Ni-NTA and a HiLoad 26/600 Superdex 200 column. The result was repeated three times. **b** Results of ITC assays performed using full-length Qki-5 with a Trx-His_6 tag (FL) indicated in (**a**) and QRE2 (UUCACUAACAA) (left) / QDE1 (right). Raw ITC data on 20 injections are shown at the top, and fitted curves are shown at the bottom. **c** Results of ITC assays performed using the STAR domain of Qki-5 with Trx-His_6 tag (S) indicated in (**a**) and QRE2 (left) / QDE1 (right). Raw ITC raw data on 20 injections are shown at the top, and fitted curves are shown at the bottom.

a

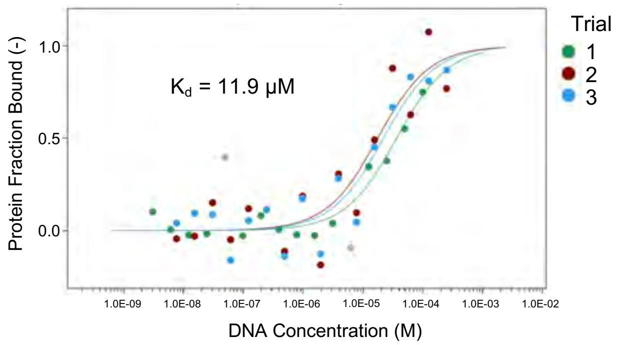
Trial
1
2
3
K_d = 11.9 μM
Protein Fraction Bound (-)
1.0
0.5
0.0
1.0E-09
1.0E-08
1.0E-07
1.0E-06
1.0E-05
1.0E-04
1.0E-03
1.0E-02
DNA Concentration (M)

b

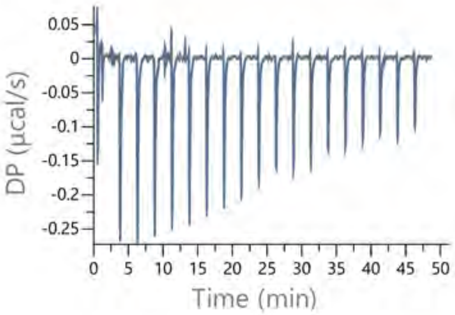
DP (μcal/s)
0.05
0
-0.05
-0.1
-0.15
-0.2
-0.25
0 5 10 15 20 25 30 35 40 45 50
Time (min)

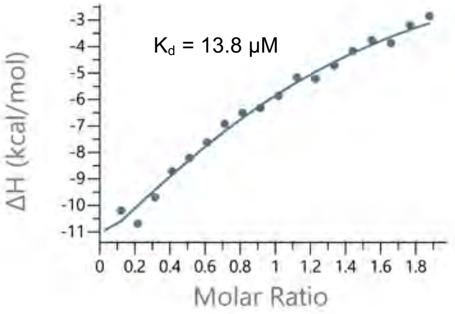
K_d = 13.8 μM
ΔH (kcal/mol)
-3
-4
-5
-6
-7
-8
-9
-10
-11
0 0.2 0.4 0.6 0.8 1 1.2 1.4 1.6 1.8
Molar Ratio

Supplementary Fig. 9: Validation of hnRNP K interaction with the known hnRNP K motif (CTCCCC) using MST and ITC assay. a Titration curve for the MST assay showing the K_d value for fluorescently labeled Trx-His_6-hnRNP K and binding motif CTCCCC. The result was repeated three times. **b** Result of ITC assay performed using Trx-His_6-hnRNP K and CTCCCC. Raw ITC data on 20 injections is shown at the top, and fitted curve is shown at the bottom.

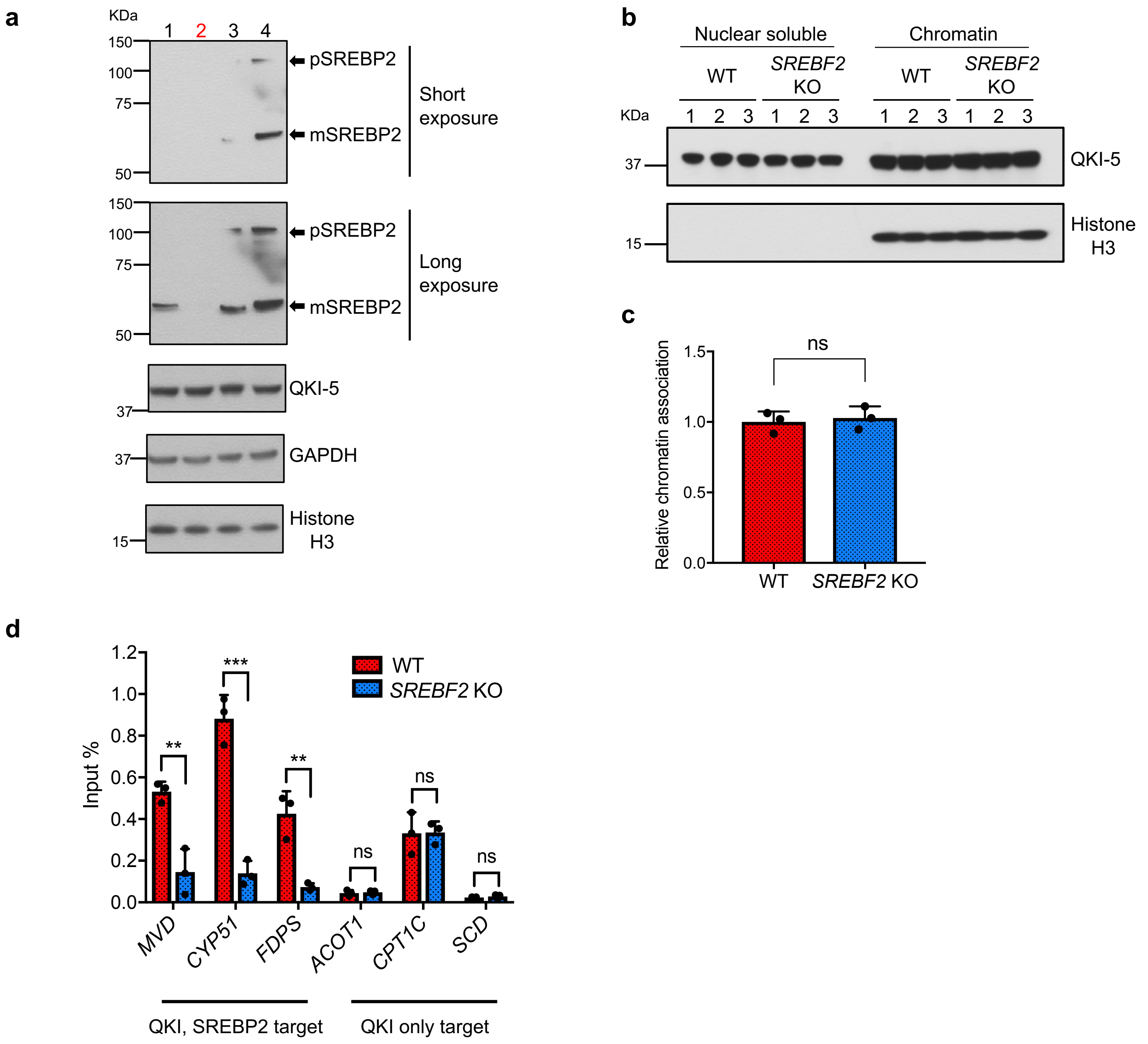

Shin et al Supplementary Fig. 10

Supplementary Fig. 10: Alteration of QKI-5 binding to chromatin upon SREBP2 depletion in the lens cells. a Immunoblots for validation of *SREBF2* deletion using the CRISPR-Cas9 system in HLE-B3 cells. The red labeled (2) indicates the *SREBF2* KO line used in the functional assay in **b-d**. pSREBP2: precursor SREBP2. mSREBP2: mature SREBP2. **b-c** Immunoblots and quantification for detection of QKI-5 in subcellular fractions of WT (n = 3) and *SREBF2* KO (n = 3) HLE-B3 cells. $p = 0.6819$, ns = not significant ($p \geq 0.05$) (two-tailed unpaired t-test). The results are presented as means with SD. Histone H3: chromatin fraction. **d** ChIP-qPCR analysis of the promoter regions of *MVD*, *CYP51*, *FDPS*, *ACOT1*, *CPT1C*, and *SCD* in WT (n = 3) and *SREBF2* KO (n = 3) HLE-B3 cells. $p = 0.005247$ (*MVD*); 0.0005638 (*CYP51*); 0.005067 (*FDPS*); 0.7016 (*ACOT1*); 0.9492 (*CPT1C*); 0.3929 (*SCD*) $^{**}p < 0.01$; $^{***}p < 0.001$; ns = not significant ($p \geq 0.05$) (two-tailed unpaired t-test). The results are presented as means with SD. All the experiments were replicated three times in the lab.

Supplementary Table 1.

Primer name	Sequence	Source
Mouse RT-qPCR primer set		
m*Hmgcs1*-Forward<br>m*Hmgcs1*-Reverse	TGGAAGCCTTTGGGGACG<br>GGTGAGTACTGTGCCAGGAC	This paper
m*Hmgcr*-Forward<br>m*Hmgcr*-Reverse	CCAAGAGAGAAAAGTTGAGG<br>TTGCACCTTTCTCTGCATTC	This paper
m*Pmvk*-Forward<br>m*Pmvk*-Reverse	AGGAGTATGCTCGGGAGCATG<br>TGTGTCACTCACCAGCCAGATAG	This paper
m*Mvd*-Forward<br>m*Mvd*-Reverse	CAGCCAATGGAGACAAGTTCC<br>GTCCTGGTCCGACCTGAGTG	This paper
m*Idi1*-Forward<br>m*Idi*1-Reverse	ATTACCTTTCCAGGTTGTTTCACC<br>TTTAGATCAACCTCTTCCAAGG	This paper
m*Fdps*-Forward<br>m*Fdps*-Reverse	TCCAGGTCCAGGACGACTACCTTG<br>CCCATAATTCTCCTCTAAGATCTGG	This paper
m*Fdft1*-Forward<br>m*Fdft1*-Reverse	GTGCTATTCCACAGGTAATG<br>GATCCGGTGATAAATCTCTTC	This paper
m*Lss*-Forward<br>m*Lss*-Reverse	GGCTCCTGGGAGGGCTCCTG<br>CTGCACAAGCAGCCCCATCTTGG	This paper
m*Cyp51*-Forward<br>m*Cyp51*-Reverse	ACTTACGACCAGTTGAAGGATCTG<br>CCTGCCACCGTCTGAGGGGTC	This paper
m*Tm7sf2*-Forward<br>m*Tm7sf2*-Reverse	CTCATCTGCCTCCTTAAGGTTATTGG<br>GGGATGGTCTCAAGACCAGCCAC	This paper
m*Msmo1*-Forward<br>m*Msmo1*-Reverse	ACGAGTTTCAGGCTCCATTTGG<br>CATAACCGCTGTGCACATCG	This paper
m*Nsdhl*-Forward<br>m*Nsdhl*-Reverse	CAGGAGAGAGCAGTACTGGATG<br>CAGGTTTTCCCCATTTCCAATC	This paper
m*Hsd17b7*-Forward<br>m*Hsd17b7*-Reverse	GAATTTCAACCAGAAGGGTCTG<br>ACAAAAAAGCGAAGGAGCCAC	This paper
m*Sc5d*-Forward<br>m*Sc5d*-Reverse	ACTTTCCAAATGGCTGGATTCATC<br>TATGTATGCGCTTGTAGACCAG	This paper

m*Actb*-Forward<br>m*Actb*-Reverse	CCACCATGTACCCAGGCATT<br>CCGATCCACACAGAGTACTT	This paper
m*Gapdh*-Forward<br>m*Gapdh*-Reverse	TCACCACCATGGAGAAGGC<br>GCTAAGCAGTTGGTGGTGCA	[1]
**Human RT-qPCR primer set**		
h*HMGCS1*-Forward<br>h*HMGCS1*-Reverse	GGGCAGGGCATTATTAGGCTAT<br>TTAGGTTGTCAGCCTCTATGTTGAA	[2]
h*HMGCR*-Forward<br>h*HMGCR*-Reverse	CCCCTCTCCAGGTGTTCACA<br>AATTGAGGTAGGTTTCATAGAGATGCT	[2]
h*IDI1*-Forward<br>h*IDI*1-Reverse	TTTCCAGGTTGTTTTACGAATACG<br>TCCTCAAGCTCGGCTGGAT	[2]
h*FDFT1*-Forward<br>h*FDFT1*-Reverse	TCAGACCAGTCGCAGTTTCG<br>CTGCGTTGCGCATTTCC	[2]
h*SQLE*-Forward<br>h*SQLE*-Reverse	CGTGCTCCTCTTGGTACCTCAT<br>CGGTCAAGGCGGAGATTATC	[2]
h*CYP51*-Forward<br>h*CYP51*-Reverse	TGCAGCCTGGCTCTTACCA<br>AGCTCTGTCCCTGCGTCTGA	[2]
h*MSMO1*-Forward<br>h*MSMO1*-Reverse	GAAAAGCCGGCACCAAGA<br>TCAAAGAGAGAATCAGCTCAAACTG	[2]
h*GAPDH*-Forward<br>h*GAPDH*-Reverse	GGATTTGGTCGTATTGGG<br>GGAAGATGGTGATGGGATT	[3]
**Mouse ChIP-qPCR primer set**		
mC-*Hmgcs1*-Forward<br>mC-*Hmgcs1*-Reverse	GGTCGGTGGCTATAAAGCTG<br>CGGGACACTCACCCAAAG	This paper
mC-*Hmgcr*-Forward<br>mC-*Hmgcr*-Reverse	GCTCGGAGACCAATAGGA<br>CCGCCAATAAGGAAGGAT	[4]
mC-*Mvk*-Forward<br>mC-*Mvk*-Reverse	AGCACGTCATCGGTGTGAT<br>TGGGAACTTCAAGCAAGCC	[5]
mC-non-target-Forward<br>mC-non-target-Reverse	ATGCCTAACTTCCAGTTCCAGG<br>AGCTTAGAGCAGAAAGCTGGT	This paper
**Human ChIP-qPCR primer set**		
hC-*HMGCR*_Forward<br>hC-*HMGCR*_Reverse	CTTATTGGTCGAAGGCTCGT<br>CTCACTAGAGGCCACCGAAC	This paper

hC-*MVD*_Forward<br>hC-*MVD*_Reverse	CACGCGCTACCACAGGATT<br>CGTCCATTGGCTGAGAGGTA	This paper
hC-*FDPS*_Forward<br>hC-*FDPS*_Reverse	GCAGGATCGCGTTATCTGTC<br>GCTCTGTGGGTCGAGTAGTT	This paper
hC-*CYP51*_Forward<br>hC-*CYP51*_Reverse	AGGTGATCTCGGCCTACATC<br>TTATTTGGGGCTGTCGGAGT	This paper
hC-non-target_Forward<br>hC-non-target_Reverse	TGCCCAGCCTCAGTTTCTTA<br>GCAACCAAACCATGAGCTGA	This paper
hC-*ACOT1*_Forward<br>hC-*ACOT1*_Reverse	GCAGACAGCTCTGCCCTAGT<br>CAAATAATCCGGCCAAACTG	This paper
hC-*CPT1C*_Forward<br>hC-*CPT1C*_Reverse	GAGAGAGGAATCGGGGTTTC<br>TATGTCCAATCCCAGTGCAA	This paper
hC-*SCD*_Forward<br>hC-*SCD*_Reverse	CCAGTCAACTCCTCGCACTT<br>AGCCGGGAATTTAAAGGCTA	This paper

Supplementary Table 2.

Cellular Pathway	-log(p-value)	p-value
Superpathway of Cholesterol Biosynthesis	9.24	5.7544E-10
RAN Signaling	7.36	4.36516E-08
Sertoli Cell-Sertoli Cell Junction Signaling	6.46	3.46737E-07
Cholesterol Biosynthesis I	5.89	1.28825E-06
Cholesterol Biosynthesis II	5.89	1.28825E-06
Cholesterol Biosynthesis III	5.89	1.28825E-06
Zymosterol Biosynthesis	5.67	2.13796E-06
Gap Junction Signaling	4.89	1.28825E-05
Germ Cell-Sertoli Cell Junction Signaling	4.57	2.69153E-05
EIF2 Signaling	4.38	4.16869E-05
VEGF Signaling	3.76	0.00017378
Regulation of eIF4 and p70S6K Signaling	3.73	0.000186209
Geranylgeranyldiphosphate Biosynthesis I	3.68	0.00020893
Amyloid Processing	3.68	0.00020893
Epithelial Adherens Junction Signaling	3.46	0.000346737
Mevalonate Pathway I	3.3	0.000501187

Supplementary Table 3.

Cellular Pathway	-log(p-value)	p-value
Protein Ubiquitination Pathway	12.6	2.51189E-13
Breast Cancer Regulation by Stathmin1	9.75	1.77828E-10
Superpathway of Cholesterol Biosynthesis	8.8	1.58489E-09
Sirtuin Signaling Pathway	7.78	1.65959E-08
EIF2 Signaling	7.74	1.8197E-08
mTOR Signaling	7.56	2.75423E-08
Geranylgeranyldiphosphate Biosynthesis I	7.44	3.63078E-08
Regulation of eIF4 and p70S6K Signaling	7.07	8.51138E-08
Glioma Signaling	6.68	2.0893E-07
Molecular Mechanisms of Cancer	6.52	3.01995E-07
Mitochondrial Dysfunction	6.44	3.63078E-07
RAN Signaling	6.27	5.37032E-07
Ephrin Receptor Signaling	5.77	1.69824E-06
Signaling by Rho Family GTPases	5.72	1.90546E-06
Gap Junction Signaling	5.63	2.34423E-06
Mevalonate Pathway I	5.57	2.69153E-06

References

1 Giulietti, A. *et al.* An overview of real-time quantitative PCR: applications to quantify cytokine gene expression. *Methods* **25**, 386-401 (2001).

2 Moon, S.-H. *et al.* p53 represses the mevalonate pathway to mediate tumor suppression. *Cell* **176**, 564-580. e519 (2019).

3 Xiang, X. *et al.* Grhl2 determines the epithelial phenotype of breast cancers and promotes tumor progression. *PloS one* **7** (2012).

4 Jeon, T.-I., Zhu, B., Larson, J. L. & Osborne, T. F. SREBP-2 regulates gut peptide secretion through intestinal bitter taste receptor signaling in mice. *The Journal of clinical investigation* **118**, 3693-3700 (2008).

5 Kim, Y.-C. *et al.* Liver ChIP-seq analysis in FGF19-treated mice reveals SHP as a global transcriptional partner of SREBP-2. *Genome biology* **16**, 268 (2015).
