# Supplementary material for: Qki activates Srebp2-mediated cholesterol biosynthesis for maintenance of eye lens transparency
Source: Nat Commun. 2021 May 21;12:3005. doi: 10.1038/s41467-021-22782-0 (PMC8139980; doi:10.1038/s41467-021-22782-0)
Supplement: Supplementary file 3 — Reporting Summary [file 41467_2021_22782_MOESM3_ESM.pdf]

nature research

Corresponding author(s): Jian Hu

Last updated by author(s): Feb 11, 2021

Reporting Summary

Nature Research wishes to improve the reproducibility of the work that we publish. This form provides structure for consistency and transparency in reporting. For further information on Nature Research policies, see our Editorial Policies and the Editorial Policy Checklist.

Statistics

For all statistical analyses, confirm that the following items are present in the figure legend, table legend, main text, or Methods section.

n/a	Confirmed	
☐	☒	The exact sample size (n) for each experimental group/condition, given as a discrete number and unit of measurement
☐	☒	A statement on whether measurements were taken from distinct samples or whether the same sample was measured repeatedly
☐	☒	The statistical test(s) used AND whether they are one- or two-sided<br>*Only common tests should be described solely by name; describe more complex techniques in the Methods section.*
☒	☐	A description of all covariates tested
☒	☐	A description of any assumptions or corrections, such as tests of normality and adjustment for multiple comparisons
☐	☒	A full description of the statistical parameters including central tendency (e.g. means) or other basic estimates (e.g. regression coefficient) AND variation (e.g. standard deviation) or associated estimates of uncertainty (e.g. confidence intervals)
☐	☒	For null hypothesis testing, the test statistic (e.g. F, t, r) with confidence intervals, effect sizes, degrees of freedom and P value noted<br>*Give P values as exact values whenever suitable.*
☒	☐	For Bayesian analysis, information on the choice of priors and Markov chain Monte Carlo settings
☒	☐	For hierarchical and complex designs, identification of the appropriate level for tests and full reporting of outcomes
☒	☐	Estimates of effect sizes (e.g. Cohen's d, Pearson's r), indicating how they were calculated

Our web collection on statistics for biologists contains articles on many of the points above.

Software and code

Policy information about availability of computer code

Data collection	Sequest (ThermoFinnigan, San Jose, CA), Image Studio (LI-CoR), DESeq2 (1.26.0)
Data analysis	Fiji-ImageJ (NIH), NanoTemper analysis software, MicroCal Origin software, GraphPad Prism 8 software, Ingenuity pathway analysis (IPA) (Qiagen Inc.), STAR (2.6.1b), Bowtie2 (2.4.1), bedtools (2.24.0), Samtools (1.10), FastQC (0.11.8), HTSeq (0.11.0), R (3.6.3), Trim Galore (v0.4.4_dev), Bowtie (v1.2.2), macs2 (v2.1.2), ngsplot, HOMER(v4.10.1)

For manuscripts utilizing custom algorithms or software that are central to the research but not yet described in published literature, software must be made available to editors and reviewers. We strongly encourage code deposition in a community repository (e.g. GitHub). See the Nature Research guidelines for submitting code & software for further information.

Data

Policy information about availability of data

All manuscripts must include a data availability statement. This statement should provide the following information, where applicable:
- Accession codes, unique identifiers, or web links for publicly available datasets
- A list of figures that have associated raw data
- A description of any restrictions on data availability

The RNA-and ChIP-seq data described in the study have been deposited in the National Center for Biotechnology Information Gene Expression Omnibus and are accessible at GSE145475 (https://www.ncbi.nlm.nih.gov/geo/query/acc.cgi?acc=GSE145475) and GSE144757 (https://www.ncbi.nlm.nih.gov/geo/query/acc.cgi?acc=GSE144757).

Field-specific reporting

Please select the one below that is the best fit for your research. If you are not sure, read the appropriate sections before making your selection.

☒ Life sciences ☐ Behavioural & social sciences ☐ Ecological, evolutionary & environmental sciences

For a reference copy of the document with all sections, see nature.com/documents/nr-reporting-summary-flat.pdf

Life sciences study design

All studies must disclose on these points even when the disclosure is negative.

Sample size	No statistical method was used to predetermine the sample size. Number of animals and biological replicates of cell lines/data were determined based on biological variation from pilot experiments.
Data exclusions	No outlier was encountered throughout the entire study.
Replication	Animal and cell line studies were performed with a minimum of three independent biological replicates. Binding affinity assays (MST and ITC) were repeated three times independently. All the attempts for data replication in the study was successful.
Randomization	There were no randomization events during the experiments for animal and cell line studies as each group was defined based on the genotype.
Blinding	There were no blinding events in the animal and cell line studies as it was sufficient to record the genotype information based on the tamoxifen-inducible system and CRISPR-Cas9 system.

Reporting for specific materials, systems and methods

We require information from authors about some types of materials, experimental systems and methods used in many studies. Here, indicate whether each material, system or method listed is relevant to your study. If you are not sure if a list item applies to your research, read the appropriate section before selecting a response.

Materials & experimental systems

n/a	Involved in the study	
☐	☒	Antibodies
☐	☒	Eukaryotic cell lines
☒	☐	Palaeontology and archaeology
☐	☒	Animals and other organisms
☒	☐	Human research participants
☒	☐	Clinical data
☒	☐	Dual use research of concern

Methods

n/a	Involved in the study	
☐	☒	ChIP-seq
☒	☐	Flow cytometry
☒	☐	MRI-based neuroimaging

Antibodies

Antibodies used	anti-Nestin (BD Biosciences, 556309), anti-GFAP (BD Biosciences, 556330), anti–Qki-5 (immunizing rabbit with a short synthetic peptide [CGAVATKVRRHDMRVHPYQRIVTADRAATGN], Genscript), anti–Qki-6 (Sigma-Aldrich, AB9906), anti-GFP (Abcam, ab13970), anti-AQP0 (Alpha Diagnostic International, AQP01-A), anti-Hmgcs1 (Abcam, ab155787), anti-Pax6 (Abcam, ab5790), anti–aB-crystallin (Abcam, ab13496), anti-ubiquitin (MBL, MK-11-3), anti-p62 (CST, 5114), anti-Hsp90 (Abcam, ab59459), anti-Hmgcr (Abcam, ab174830), anti-Fdps (Abcam, ab189874), anti–aA-crystallin (Abcam, ab5595), anti–b-crystallin (Santa Cruz Technology, sc-22745), anti–b-actin (Sigma-Aldrich, A5441), anti-Srebp2 (10007663, Cayman Chemical), normal rabbit immunoglobulin G (CST, 2729), anti-SREBP2 (Abcam, ab30682), anti-GAPDH (Santa Cruz Biotechnology, SC-32233), anti-SP1 (Abcam, ab13370), anti-Histone H3 (Abcam, ab5176), anti-Pol II (Abcam, ab817), anti-Flag (Sigma-Aldrich, 1804), anti-HA (Abcam, ab1818), HRP-conjugated secondary antibodies (Thermo Fisher, anti-mouse (31320), ant-rabbit (A16104))
Validation	anti-Nestin and anti-GFAP antibodies were validated by immunoflorescence (IF) by Takashi Shingu et al., Nat Genet., 2017, Jan;49(1):75-86. anti–Qki-5 antibody was validated by IF, western blot (WB), immunoprecipitation (IP), and chromatin immunoprecipitation (ChIP) by Xin Zhou et al., J Clin Invest. 2020 May 1;130(5):2220-2236. anti–Qki-6 antibody was validated by IF, WB, and IP by the supplier and Ruben G de Bruin et al., Sci Rep., 2016 Feb 24;6:21643. anti-GFP antibody was validated by IF by the supplier. anti-AQP0 antibody was validated by IF by Rosica S Petrova et al., Exp Eye Res. 2015 Mar;132:124-35. anti-Hmgcs1 antibody was validated by IF and WB by the supplier. anti-Pax6 antibody was validated by IF by the supplier. anti–aB-crystallin was validated by IF and WB by the supplier. anti–aA-crystallin and anti–b-crystallin antibodies were validated by WB by the supplier. anti-ubiquitin was validated by WB by the supplier. anti-p62 was validated by WB by Takashi Shingu et al., Nat Genet., 2017, Jan;49(1):75-86. anti-Hsp90 was validated by WB by the supplier. anti-Hmgcr was validated by WB by the supplier. anti-Fdps was validated by WB by the supplier. anti-b-actin was validated by WB by the supplier. anti-Srebp2 was validated by WB, IP, and ChIP by Ayano Kondo et al., Cell Rep. 2017 Feb 28;18(9):2228-2242 and the supplier. Normal rabbit immunoglobulin G was validated by IP by the supplier. anti-SREBP2 was

validated by WB by the supplier. anti-GAPDH was validated by WB by the supplier. anti-SP1 was validated by WB by the supplier. anti-Histone H3 was validated by WB by the supplier. anti-Pol II was validated by ChIP by the supplier. anti-Flag and anti-HA were validated by WB by the supplier. HRP-conjugated secondary antibodies were validated by WB by Takashi Shingu et al., Nat Genet., 2017, Jan;49(1):75-86.

Eukaryotic cell lines

Policy information about cell lines

Cell line source(s)	HLE-B3 cell line obtained from ATCC (Cat# CRL-11421) and HEK293T cell line
Authentication	HLE-B3 cell line: Generated by U P Andley et al. Propagation and immortalization of human lens epithelial cells in culture. Invest. Ophthalmol. Vis. Sci. 35: 3094-3102, 1994. PubMed: 8206728<br>HEK293T cell line: Validated by Xin Zhou et al., Mature myelin maintenance requires Qki to coactivate PPARβ-RXRα-mediated lipid metabolism. J Clin Invest. 2020 May 1;130(5):2220-2236. doi: 10.1172/JCI131800. PMID: 32202512
Mycoplasma contamination	Mycoplasma testing was confirmed negative in HLE-B3 cell line and HEK293T cell line.
Commonly misidentified lines (See ICLAC register)	No commonly misidentified cell lines were used in the study.

Animals and other organisms

Policy information about studies involving animals; ARRIVE guidelines recommended for reporting animal research

Laboratory animals	Mouse, C57BL/6, male and female, age postnatal 17-30 days, Temperature: 72F, Humidity: 50%
Wild animals	No wild animals were used in the study.
Field-collected samples	No field collected samples were used in the study.
Ethics oversight	All mouse experiments were conducted in accordance with protocols approved by the Institutional Animal Care and Use Committee of The University of Texas MD Anderson Cancer Center.

Note that full information on the approval of the study protocol must also be provided in the manuscript.

ChIP-seq

Data deposition

☒ Confirm that both raw and final processed data have been deposited in a public database such as GEO.

☒ Confirm that you have deposited or provided access to graph files (e.g. BED files) for the called peaks.

Data access links<br>*May remain private before publication.*	GSE144757 (https://www.ncbi.nlm.nih.gov/geo/query/acc.cgi?acc=GSE144757)
Files in database submission	HLE-B3_WT_QKI-5<br>HLE-B3_WT_SREBP2<br>HLE-B3_QKI KO_SREBP2<br>HLE-B3_WT_POL II<br>HLE-B3_QKI KO_POL II<br>HLE-B3_WT_Input<br>HLE-B3_QKI KO_Input<br>NLPC_WT_Qki-5<br>NLPC_WT_Srebp2<br>NLPC_Qk-/-_Srebp2<br>NLPC_WT_Pol II<br>NLPC_Qk-/-_Pol II<br>NLPC_WT_Input<br>NLPC_Qk-/-_Input<br>NLPC_WT_IgGR<br>NLPC_WT_Qki-5_rep2<br>HLE-B3_WT_QKI-5_rep2
Genome browser session (e.g. UCSC)	NA

Methodology

Replicates	Two replicates for HLE-B3_WT_QKI-5 and NLPC_WT_Qki-5, one replicate for the others.
Sequencing depth	20-30 million paired-reads for each sample.

Antibodies	anti–Qki-5 (immunizing rabbit with a short synthetic peptide [CGAVATKVRRHDMRVHPYQRIVTADRAATGN], Genscript), anti-Srebp2 (10007663, Cayman Chemical), anti-Pol II (Abcam, ab817), or normal rabbit IgG (CST, 2729) antibodies
Peak calling parameters	Reads were mapped to the mouse genome (mm10 version) or human genome (hg19 version) using Bowtie (v1.2.2) with the parameters '-M 1 --best --strata'. Peaks were called by macs2 (v2.1.2) with the parameters 'macs2 callpeak -f BAM -g mm/hs -q 0.05 -t ChIP.bam -n NAME -c INPUT.bam' or Homer (v4.10.1) with the following steps: makeTagDirectory and findPeaks Sample_tag -style factor -size auto -minDist default -i Input_tag -fdr 0.001.
Data quality	Peaks were filtered by FDR 0.05 or 0.001.
Software	Galore (v0.4.4_dev) , Bowtie (v1.2.2), macs2 (v2.1.2), Homer (v4.10.1), samtools (v1.8) and depptools (v3.3.0).
